# A Tiny Change Makes a Big Difference in the Anti-Parasitic Activities of an HDAC Inhibitor

**DOI:** 10.3390/ijms20122973

**Published:** 2019-06-18

**Authors:** Corinne Loeuillet, Bastien Touquet, Jean François Guichou, Gilles Labesse, Denis Sereno

**Affiliations:** 1 Univ Grenoble Alpes, CNRS, CHU Grenoble Alpes, Grenoble INP, TIMC-IMAG, F-38000 Grenoble, France; corinne.loeuillet@univ-grenoble-alpes.fr; 2 Institute for Advanced Biosciences (IAB), Team Host-Pathogen Interactions & Immunity to Infection, INSERM U 1209, CNRS UMR 5309, Univ Grenoble Alpes, F-38000 Grenoble, France; bastien.touquet@univ-grenoble-alpes.fr; 3 Centre de Biochimie Structurale (CBS), INSERM, CNRS, Univ Montpellier, 34000 Montpellier, France; 4 Institute of Research for the Development (IRD), Univ Montpellier, MiVegec, 34000 Montpellier, France; 5 Institute of Research for the Development (IRD), Univ Montpellier, InterTryp, 34000 Montpellier, France

**Keywords:** HDACi, *Toxoplasma*, Hydroxamate, In silico modeling

## Abstract

We previously synthesized an hydroxamate derivative (N-hydroxy-4-[2-(3-methoxyphenyl)acetamido]benzamide) named 363 with potent anti-*Toxoplasma gondii* activity and histone deacetylase inhibitor (HDACi) effects. Here we show that 1-N-hydroxy-4-N-[(2-methoxyphenyl)methyl]benzene-1,4-dicarboxamide, a 363 isomer, does not have antiparasitic potency and has a 13-fold decrease in HDACi activity. The in silico modeling of *T. gondii* HDACs of the type II strain discloses identity varying from 25% to 62% on more than 250 residues for S8EP32_TOXG and A0A125YPH4_TOXGM. We observed a high conservation degree with the human HDAC2 (53% and 64% identity, respectively) and a moderate one with the human HDAC8 (30–40%). Two other TgHDACs, S8F6L4_TOXGM and S8GEI3_TOXGM, were identified as displaying a higher similarity with some bacterial orthologs (~35%) than with the human enzymes (~25%). The docking in parallel of the two compounds on the models generated allowed us to gain insights on the docking of these hydroxamate derivatives that guide their specificity and potency against *T. gondii* histone deacetylase. This information would constitute the rationale from which more specific derivatives can be synthetized.

## 1. Introduction

About one-third of the world’s human population is infected with *Toxoplasma gondii* [1]. *Toxoplasma* infection in humans occurs via the ingestion of tissue cysts with raw or undercooked meat or by consumption of oocysts with contaminated food, water, vegetables, fruits, etc. Congenital transmission from mother to fetus is also possible when a woman gets an infection during pregnancy [2]. *Toxoplasma* infection, which is usually asymptomatic in immunocompetent individuals, can be threatening in immunocompromised or congenitally infected patients. High odds ratios (ORs) of *Toxoplasma* infection are reported in HIV/AIDS patients in Asia and Africa and in cancer patients in Asia [3].

Pyrimethamine (PYR) and sulfadiazine (SDZ) are used for treatment or prophylaxis of toxoplasmosis, but these drugs have severe side effects (neutropenia, leucopenia, severe platelet count decrease, thrombocytopenia, and hypersensitivity reactions). Other molecules, like azithromycin, clarithromycin, spiramycin, atovaquone, dapsone, and cotrimoxazole (trimethoprim-sulfamethoxazole) have also been used, with limited efficiency because these molecules have no effect on the bradyzoite form of the parasite [4]. Finally, it appears that *Toxoplasma* drug resistance is ongoing, urging the search for novel drug targets and new chemotherapies with novel mechanisms of action [5].

A fungal metabolite, apicidin, exhibits nanomolar histone deacetylase inhibitor (HDACi) activity and exerts a high anti-*Plasmodium falciparum* activity [6]. Histone deacetylases (HDACs) play key roles in diverse intracellular processes and epigenetic regulation, through the modification of histone and non-histone proteins to repress transcription. In human cells, 18 HDACs have been identified [7] and are classified according to their sequence homology to yeast proteins and their dependency on either zinc or NAD^+^ as the co-factor [8]. Evidence pinpoints that zinc- or NAD-dependent HDACs are promising drug targets in a wide variety of parasitic diseases, including schistosomiasis, malaria, leishmaniasis, trypanosomiasis, and toxoplasmosis ([9,10,11,12,13,14,15,16,17,18,19,20,21] and reviewed in [22]). 

We recently synthetized hydroxamate derived compounds and investigated their anti-Trypanosomatids, anti-*Toxoplasma gondii* activities in link with their HDAC inhibitory potency [23]. Here, we further address the HDAC inhibitory potency of an isomer of our best performing compound to interfere with the multiplication of *T. gondii* in relation with its HDACi activity.

## 2. Results and Discussion

### 2.1. HDACi Activity and Anti-*T. gondii* Effect

**Figure 1:**
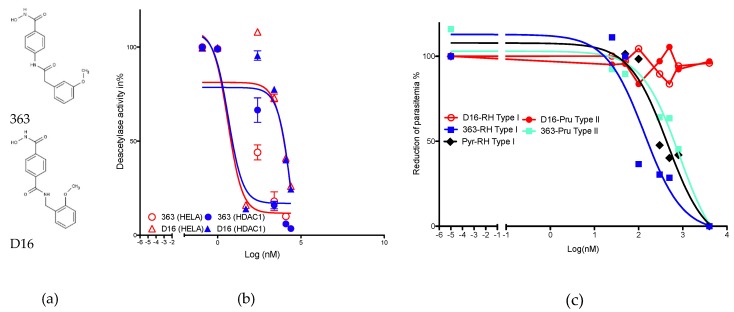
Hstone deacetylase inhibitor (HDACi) and anti-*Toxoplasma gondii* activity of the compounds. Structure of 363 and D16 (**a**), deacetylase inhibitory activity (**b**), and anti-parasitic activity against RH type I and Pru type II strains of *T. gondii*. Pyrimethamine (Pyr) was used as a positive control of inhibition (**c**). Results are representative of one experiment performed in triplicates.

Compound 363 (N-hydroxy-4-[2-(3-methoxyphenyl)acetamido]benzamide) exerts potent histone deacetylase inhibitory activity recorded in HeLa cell nuclear extract, which contains mainly HDACs 1, 2, 6, and 8 (Figure 1A). We measured an IC_50_ of 495 +/– 66 nM. By contrast, D16, 1-N-hydroxy-4-N-[(2-methoxyphenyl)methyl]benzene-1,4-dicarboxamide, (Figure 1A) shows a lower efficiency in inhibiting deacetylase activity of HeLa nuclear extracts (IC_50_ of 6683 +/– 865 nM). The 363 isomer is thus 13-fold less efficient in HDACi activity measured with HeLa nuclear extracts. Deacetylase activity of the recombinant HDAC1 enzyme confirms the lower potency of D16 as compared to 363 (Figure 1B). We previously documented the capacity of 363 to inhibit type I and II strains of *T. gondii* with IC_50_ of 350 and 2270 nM, respectively [23] and reported that type I strains of *T. gondii* are 6-fold more susceptible than type II ones [23]. Here, we recorded an IC_50_ below 1000 nM for type I (153 nM) or type II (853 nM) strains of *T. gondii*, that confirm that type I strains are 5.5 fold more susceptible than type II ones. Pyrimethamine, an anti-*T. gondii* compound in commercial use, exerts a strong activity with an IC_50_ of 453 nM. No anti-parasitic activity is recorded for the compound D16 even at concentrations higher than 1000 nM (Figure 1C).

### 2.2. Molecular Docking 

**Figure 2:**
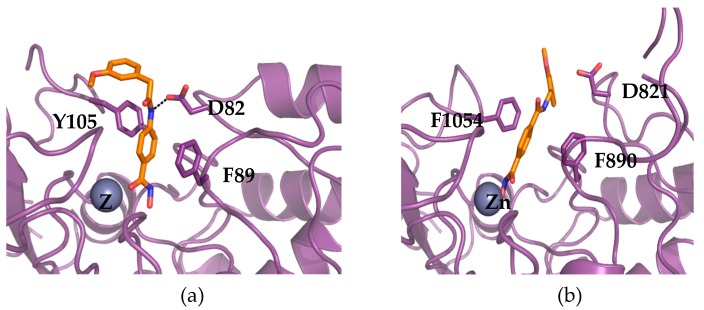
Docking of 363 and D16 in the models of TgHDACs. Model of S8GEI3_TOXGM from *T. gondii* M49 in complex with ligand 363 (**a**). Model of the same target from *T. gondii* in complex with ligand D16 (**b**). Both models were produced using the server @TOME-2 and their active site delineated around the co-crystallized ligand of the templates. Ligands were docked in 5–10 distinct models by the software PLANTS using the co-crystallized ligand as a shape restraint. The most representative configurations were selected and correspond to models based on the templates PDB5WGM and PDB5G1A for 363 and D16, respectively. The putative binding site of the zinc atom is indicated by a grey sphere labeled with the symbol Zn. The chelating sidechains are not shown for clarity. On the contrary, the two phenylalanine (F890 and F1054) and the aspartate (D821) residues interacting directly with the benzohydroxamate moieties are shown (in stick). The two ligands are indicated in orange stick and the protein backbone in violet ribbon. The only hydrogen bond shown is with this aspartate (black dashed line) and it is predicted to discriminate between compound 363 and D16. The figure was drawn using pymol (www.pymol.org).

Besides one enzyme that appears to be truncated, we modeled with a fair confidence four HDACs of *T. gondii*. All the results for the HDACs from *T. gondii* can be found in the following web page and the links therein: http://atome4.cbs.cnrs.fr/htbin-post/ATOME_V3/SUPERATOME/aff_study_stat_base.cgi?M=W&WD=ATOME_V3/HDAC_ME49_270219. The sequence identity of these HDACs ranges from 25% to 62% in over more than 250 residues for S8EP32_TOXG and A0A125YPH4_TOXGM; conservation is high with the human HDAC2 (53% and 64% identity, respectively) and moderate with the human HDAC8 (30–40%). This led to theoretical models with acceptable quality (QMEAN ~0.3). S8F6L4_TOXGM and S8GEI3_TOXGM show stronger similarity with some bacterial orthologs (~35%) than with the human enzymes (~25%). For the former, the overall quality of the models is lower because of difficulty to align some regions that led to missing parts in our model. In all cases, the active sites can be reasonably well modeled. Using co-crystallized ligands as pharmacophoric anchors, the two chemical compounds (363 and D16) were docked in parallel on several models. For three HDACs (A0A125YPH4_TOXGM, S8EP32_TOXGM, and S8GEI3_TOXGM), the best models were selected based on sequence identity and coverage. Only five reliable models could be gathered for the fourth one (S8F6L4_TOXGM); nevertheless, the number of pharmacophoric anchors was increased to four (instead of two for the other targets). The 20 predicted configurations were clustered by similarity and evaluated by various mean, scoring function, and visualization surveys. While the affinity prediction barely discriminates between the two ligands, a global trend can be deduced. The overall docking configurations are very similar; both compounds show interaction with the zinc atom of the enzymes with their common hydroxamate moiety, the phenyl group of the compounds that is linked to the hydroxamate being stacked by two phenylalanines (see Figure 2). 

In compound 363, the amide group often forms a favorable hydrogen bond with an aspartate conserved in many HDACs, while its second phenyl group lies on the surface of the protein and interacts with the hydrophobic residues of the enzyme side chains (which vary from one model to another). On the contrary, for D16, the hydrogen bond frequently recorded for 363, is less often present and corresponds with a conformation of D16 that brings the oxygen atom of the methoxy group close to the oxygen of the amide group. This unfavorable conformation would decrease the overall affinity of the compound, without interfering directly with the predicted affinity results that are based only on protein–ligand interactions. Accordingly, the two substitutions that differentiate D16 from 363 may antagonize with the proper binding of D16 into the active site of *T. gondii* HDACs. Unfortunately, the quality of the models we gather does not allow finer analysis and prevents any further predictions of the actual affinity profile. While the lower affinity for the putative targets can be predicted by similarity with the lower activity observed against human orthologs (see HDAC1) and the theoretical models discussed above, the low activity of D16 compared to the parent (363) can also be due to a poorer cell penetration.

## 3. Materials and Methods 

### 3.1. General Procedures for the Synthesis of Derivatives 

Compounds 363 (N-hydroxy-4-[2-(3-methoxyphenyl)acetamido]benzamide) and D16 (1-N-hydroxy-4-N-[(2-methoxyphenyl)methyl]benzene-1,4-dicarboxamide), represented in Figure 1A, were synthetized according to the general procedure previously described [23]. Synthetic schemes for D16 and 363 synthesis can be found in supplementary data Supplementary S1. Mass spectroscopic and purity data, with HPLC can be found in supplementary data Supplementary S2.

### 3.2. Antiparasitic Activity and Cytotoxicity.

Confluent Human Foreskin Fibroblast (HFF) monolayers were infected with YFP-type I Rh kindly provided by B. Striepen [24] or Tomato-type II Pru kindly provided by Professor N. Blanchard (Toulouse), at 5 × 10^4^ parasites per well. They were then centrifuged for 30 s at 250 *g* and incubated for 30 min in a water bath at 37 °C to allow invasion. The wells were washed three times with PBS to eliminate extracellular parasites, and the drugs were added at concentrations ranging from 0.01 to 3.0 μM. After incubation for 24 h at 37 °C in a humidified atmosphere containing 5% CO_2_, the cells were fixed in 2.5% methanol-free formaldehyde (Tebu-bio, Le Perray-en-Yvelines, France)/PBS for 30 min at room temperature. Nuclei were stained with Hoechst 33258 (2 µg/mL, Molecular Probes) for 20 min and washed three times with water. The number of infected cells, i.e., cells harboring a parasitophorous vacuole (see supplementary data S3 for an example), and the number of parasites per vacuole were determined using an Olympus ScanR microscope (×20 objective) and ScanR software. The parasitic index in % compared to the control was calculated as follows: [Parasitic index (PI) = ((number of parasite/100 cells in treated wells) × (% of infected macrophage in treated well)/(number of parasite/100 cells in untreated wells) × (% of infected macrophage in untreated wells)) × 100]. 

### 3.3. HDAC Assays

The HDACi effect against HeLa nuclear extracts and recombinant proteins were measured using a fluorometric HDAC assay kit (Active Motif, La Hulpe, Belgium) according to the manufacturer’s instructions. Briefly, 30 μL of HeLa nuclear extract (Active Motif, Belgium) or HDAC 1 recombinant protein (Active Motif, Belgium) were mixed with 5 μL of a 10× compound and 10 μL of assay buffer. A positive control, containing trichostatin A at a final concentration of 2 μM, was analyzed for each independent experiment. A fluorogenic substrate (10 μL) was added, and the reaction was allowed to proceed for 30 min at room temperature and then stopped by the addition of a developer containing trichostatin A. The fluorescence was monitored after 30 min at excitation and emission wavelengths of 360 and 460 nm, respectively.

### 3.4. Comparative Modeling and Ligand Docking 

Protein sequences were recovered from the database UNIPROT for the *Toxoplasma* strain Me49 as representative type II *T. gondii*. Three-dimensional models were built using SCWRL 3.0 [25] as implemented in the server @TOME [26]. One sequence (S8GBW3_TOXGM) appeared truncated in C-terminus and may correspond to a pseudogene. The 10 best models were selected based on sequence identity and coverage for three other sequences (A0A125YPH4_TOXGM, S8EP32_TOXGM and S8GEI3_TOXGM), while only five reliable models could be gathered for the fourth one (S8F6L4_TOXGM). Then, active site boundaries in each structural model were deduced as the vicinity of the co-crystallized ligand as well as hydroxamate compounds transferred from related HDACs by protein–protein superposition using the @TOME-2 comparative option. The same chemical entities served, in addition, to define a shape restraint to guide the docking in the automatically computed models. Only compounds harboring a hydroxamate moiety were considered here. This pharmacophoric feature compensates for the rather low overall sequence similarity (25–35%) for A0A125YPH4_TOXGM (62% identical to human HDAC2) as well as the absence of the zinc cation in the modeled binding sites. Finally, the two inhibitors were docked into the various active sites using PLANTS [27] as implemented in the server @TOME2. The best configurations were selected by visualization using the JavaScript applet JSMol (www.jmol.org).

## 5. Conclusions

Altogether, these results confirm the specificity of our best-performing compound in acting as an anti-toxoplasma compound associated with high HDACi potency. It is now necessary to synthetize new derivatives of our lead compound in order to gain in anti-*T. gondii* activity but also to increase the specificity and activity against *T. gondii* HDACs. 

## Supplementary Materials

Supplementary materials can be found at https://www.mdpi.com/1422-0067/20/12/2973/s1.

## Abbreviations

HDAC	Histone deacetylase
HIV/AIDS	Human Immunodeficiency Viruses/Acquired ImmunoDeficiency Syndrome
HFF	Human Foreskin Fibroblast
NAD	Nicotinamide Adenine Dinucleotide
PYR	Pyrimethamine
SDZ	Sulfadiazine
