# Supplementary material for: A Tiny Change Makes a Big Difference in the Anti-Parasitic Activities of an HDAC Inhibitor
Source: Int J Mol Sci. 2019 Jun 18;20(12):2973. doi: 10.3390/ijms20122973 (PMC6627744; doi:10.3390/ijms20122973)
Supplement: Supplementary file 1 [file ijms-20-02973-s001.zip › ijms-521005 supplementary/SuppS1.pdf]

Data S1, Synthesis schemes of the compounds.

Synthesis of compounds 363 and D16 was performed as describe and the synthetic scheme is given in Scheme 1-2. Amine derivative (1 eq.) was dissolved in dry DCM (0.2 M). Triethylamine (3 eq.) and the acid chloride derivative (1 eq.) were added successively at room temperature. After the reaction was complete (TLC control), the reaction mixture was concentrated. The residue was taken up with EtOAc and the organic phase was washed with a solution of 1 M HCl, a saturated solution of $NaHCO_3$ and brine, dried over Na_2SO_4, filtrated and concentrated to afford the amide derivatives. The products were used whitout any purification in the next step. Amide derivatives (1 eq.) were dissolved in THF (3 ml). A solution of LiOH (3 eq.) in 3 ml of water was added and the reaction mixture was heated at 40 °C overnight. The reaction mixture was concentrated. The residue was taken up with 30 ml of water and the aqueous phase was washed with 20 ml of EtOAc, then the aqueous phase was acidified to pH 2 with a solution of 1 M HCl. The aqueous phase was extracted three times with 20 ml of EtOAc. The organic phases were combined and dried over Na_2SO_4, filtrated and concentrated to afford the acid derivatives. Acids derivatives (1 eq.) were dissolved in dry DMF (5 ml). Ethyl chloroformate (1.2 eq.) and N-methyl-morpholine (1.3 eq.) were added successively at 0 °C. After 10 minutes, a solution of hydroxylamine (2 eq.) in MeOH (10 ml) was added and the reaction mixture was warm up to room temperature. After 15 hours, the reaction mixture was concentrated. The residue was taken up with EtOAc and the organic phase was washed with a saturated solution of $NaHCO_3$ and brine, dried over Na_2SO_4, filtrated and concentrated. The crude products were purified by flash chromatography to afford the hydroxamate derivatives. The characterization data of compound **363** and **D16** were given a follows and HPLC, MS and NMR spectra can be found in Supplementary data.

Scheme 1: **Synthetic routes of 363.** Reagents and conditions: (a) m-OMePh-CO-Cl, NEt_3, DCM; (b) LiOH, THF/H_2O 40 °C; (c) (i) Cl-CO_2Et, NMM, DMF, (ii) NH_2OH, MeOH.

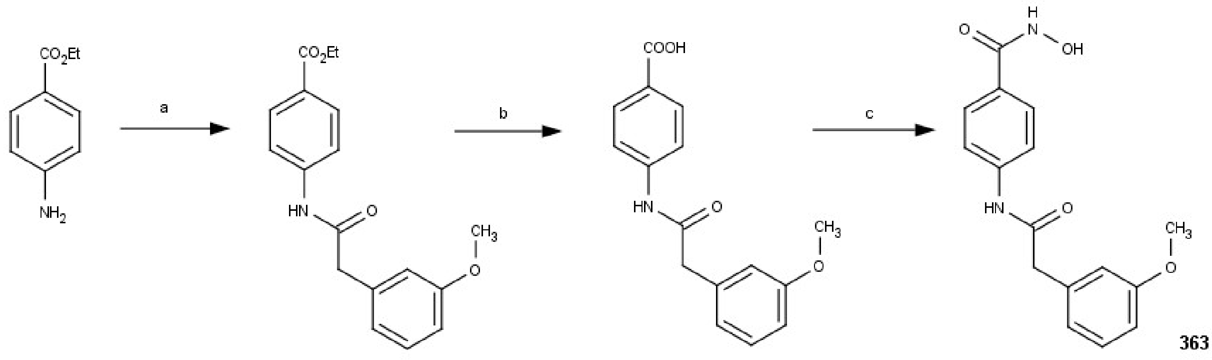

363

Scheme 2: **Synthetic routes of D16.** Reagents and conditions: (a) o-OMePh-CH_2NH_2, NEt_3, DCM; (b) LiOH, THF/H_2O 40 °C; (c) (i) Cl-CO_2Et, NMM, DMF, (ii) NH_2OH, MeOH.

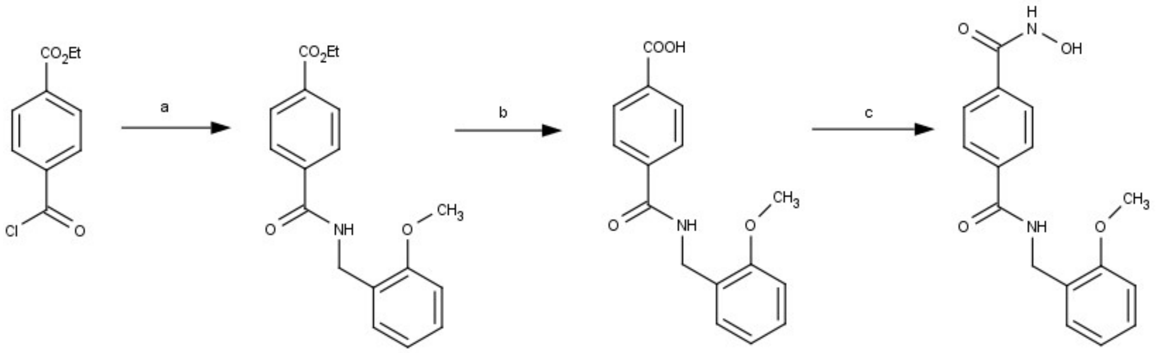

D16

N-hydroxy-4-[2-(3-methoxyphenyl)acetamido]benzamide (363)

^{1}H NMR (DMSO-d6) δ 11.09 (s, 1H), 10.35 (s, 1H), 8.93 (s, 1H), 7.70 (d, *J* = 5.8 Hz, 2H), 7.64 (d, *J* = 5.8 Hz, 2H), 7.22 (t, *J* = 5.4 Hz, 1H), 6.89 (m, 2H), 6.82 (d, *J* = 4.8 Hz, 1H), 3.74 (s, 3H), 3.63 (s, 2H). HPLC purity 99%. MS ESI+H^+ calc. 301.32 exp. 301.10.

1-N-hydroxy-4-N-[(2-methoxyphenyl)methyl]benzene-1,4-dicarboxamide (D16)

^{1}H NMR (DMSO-d6): d 11.31 (br s, 1H); 9.14 (br s, 1H); 8.95 (t, *J*=5.8Hz, 1H); 7.96 (d, *J*=8.5Hz, 2H); 7.83 (d, *J*=8.5Hz, 2H); 7.24 (dt, *J*=7.5Hz and 1.4Hz ,1H); 7.18 (dd, *J*=7.4 and 1.4Hz, 1H); 6.99 (dd, *J*=7.5 and 0.8Hz, 1H); 6.90 (dt, *J*=7.4 and 0.8Hz, 1H); 4.46 (d, *J*=5.8Hz, 2H); 3.83 (s, 3H). HPLC purity 100%. MS ESI+H^+ calc. 301.32 exp. 301.40.
