# Supplementary material for: A Tiny Change Makes a Big Difference in the Anti-Parasitic Activities of an HDAC Inhibitor
Source: Int J Mol Sci. 2019 Jun 18;20(12):2973. doi: 10.3390/ijms20122973 (PMC6627744; doi:10.3390/ijms20122973)
Supplement: Supplementary file 1 [file ijms-20-02973-s001.zip › ijms-521005 supplementary/SuppS2.pdf]

High pressure chromatography, mass spectrometry and nuclear magnetic reasonance of 363 (A) and D16 (B).

A

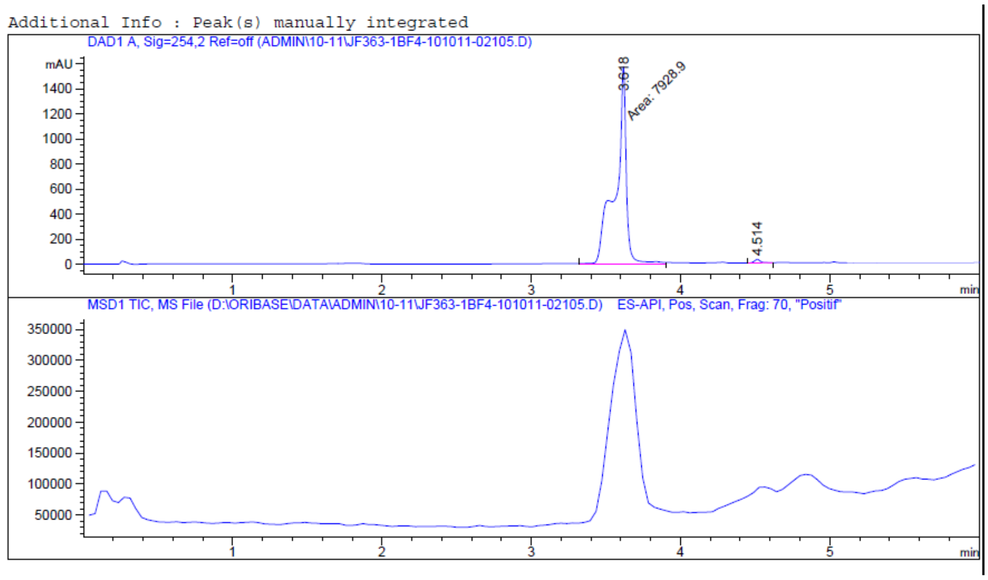

High Pressure Liquid Chromatography (HPLC) of **363**

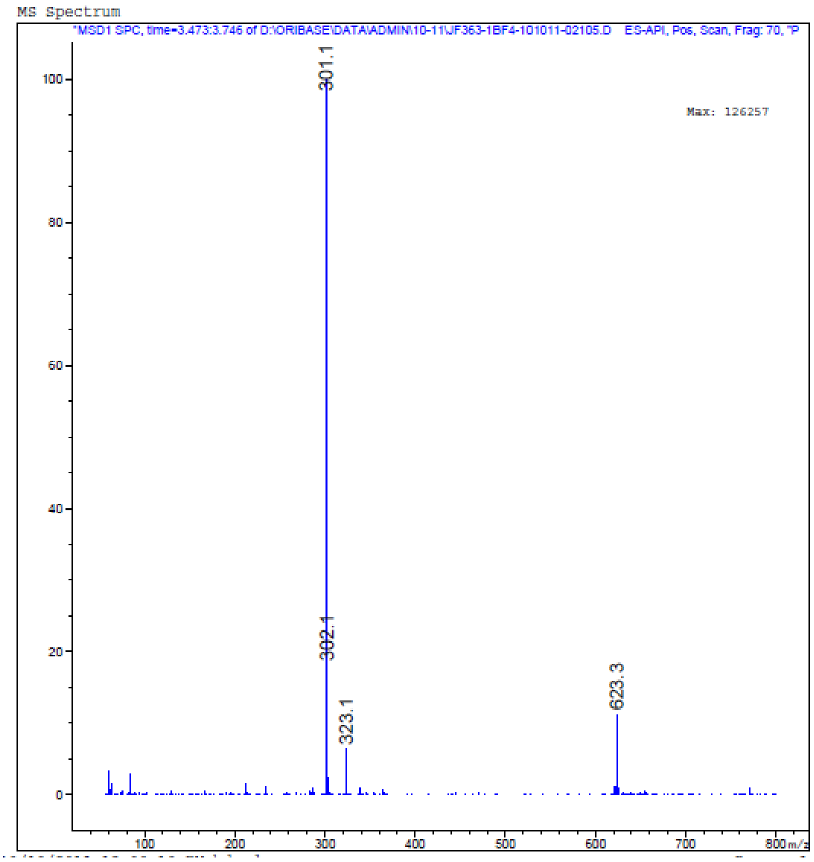

Mass Spectrometry (MS) of **363**

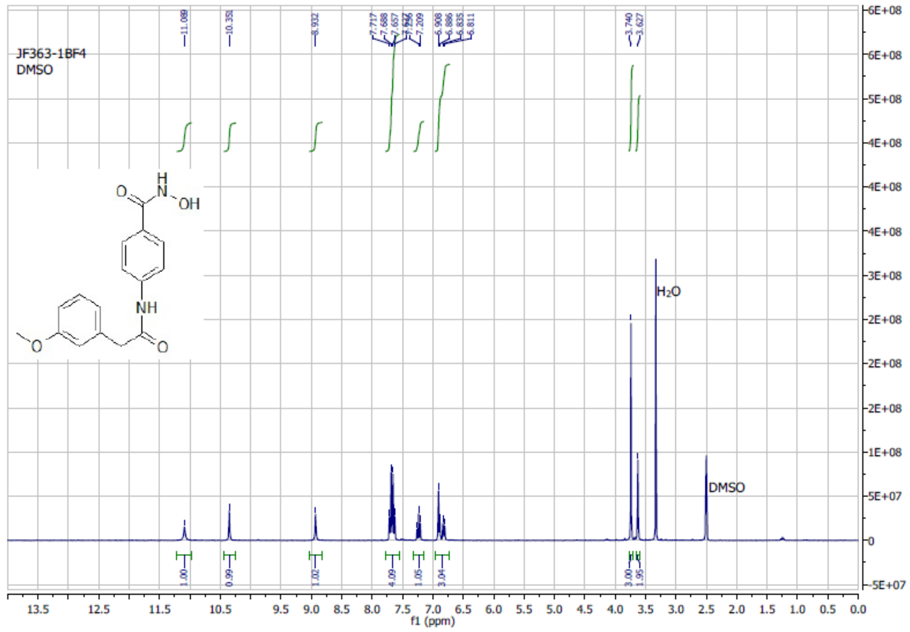

Nuclear Magnetic Resonance (NMR) of **363**

B

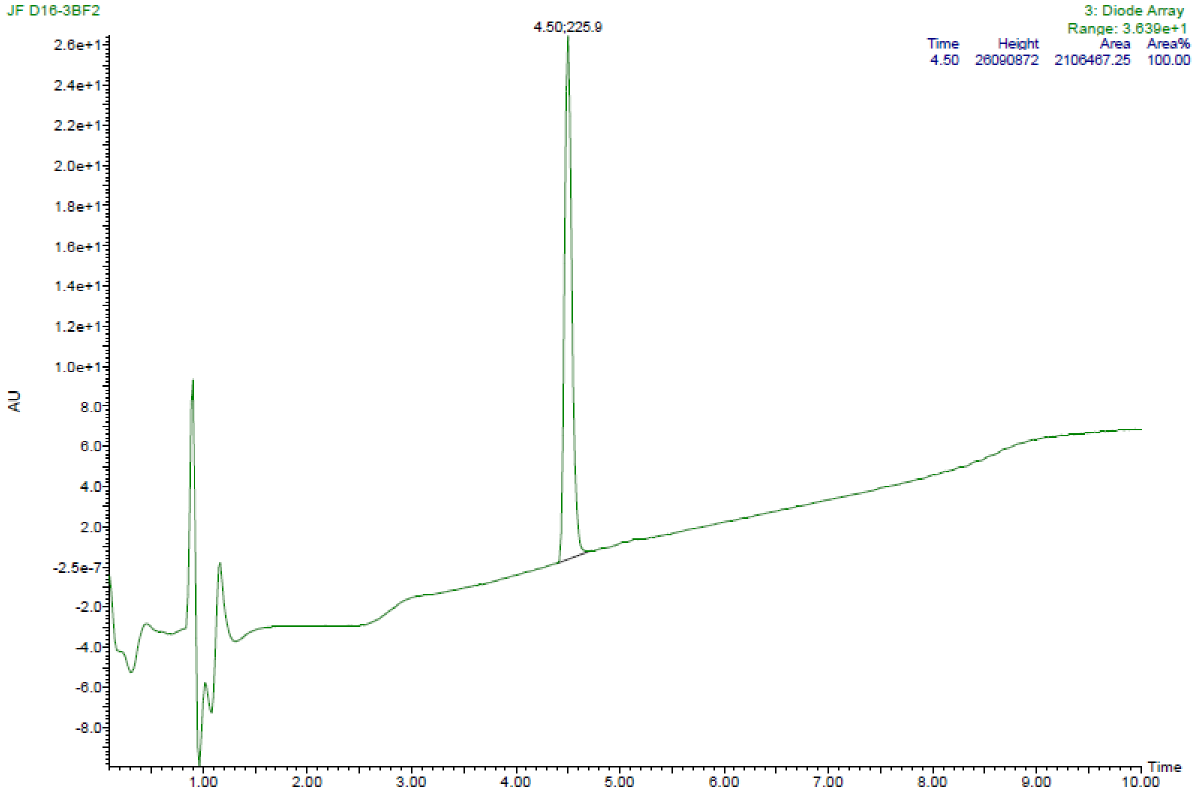

High Pressure Liquid Chromatography (HPLC) of **D16**

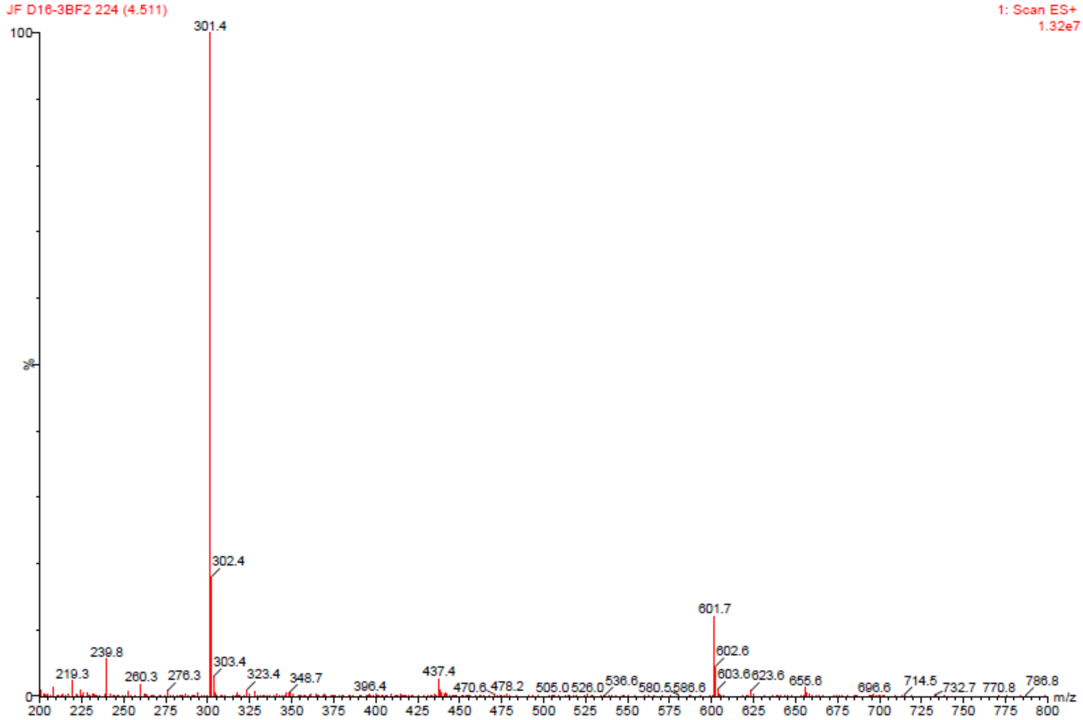

Mass Spectrometry (MS) of **D16**

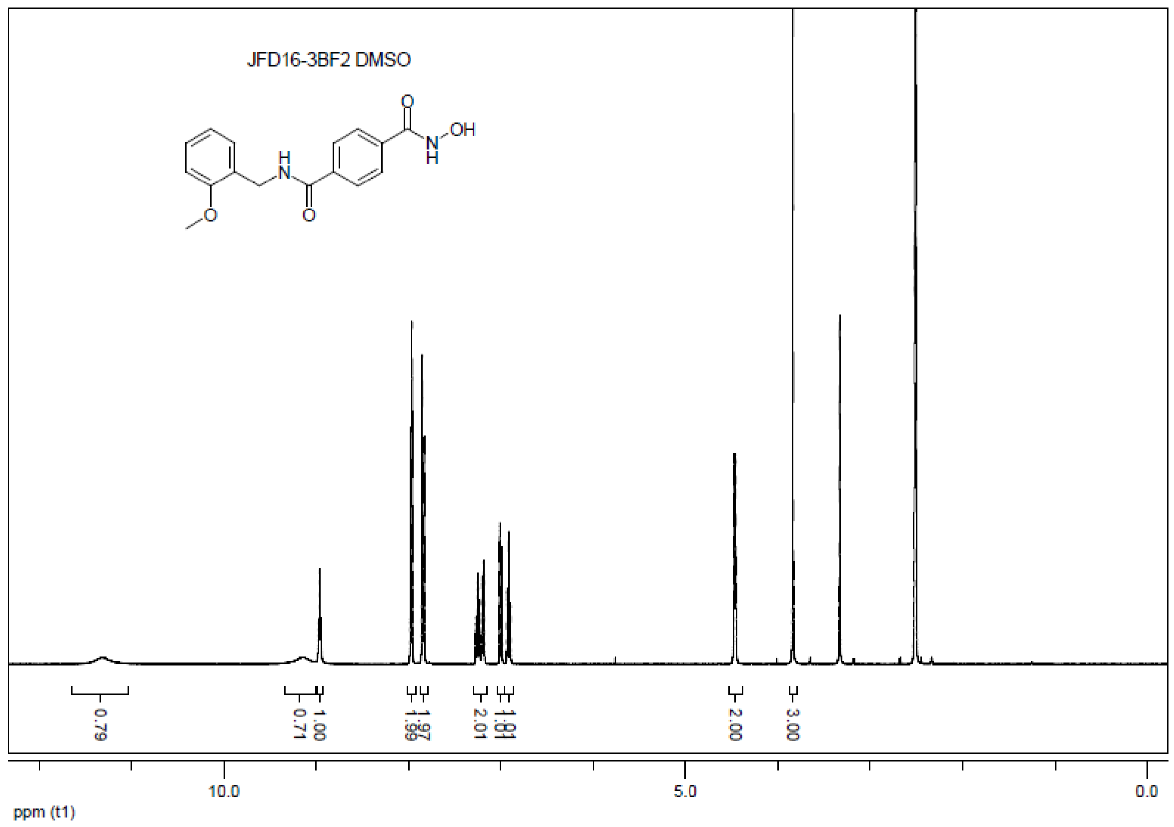

Nuclear Magnetic Resonance (NMR) of **D16**
